# Epithelial-myoepithelial carcinoma: a population-based survival analysis

**DOI:** 10.1186/s12901-018-0063-2

**Published:** 2018-08-16

**Authors:** Mitchell R. Gore

**Affiliations:** Department of Otolaryngology, SUNY Upstate Medical University, Physicians Office Building North, Suite 4P, 4900 Broad Road, Syracuse, NY 13215 USA

**Keywords:** Epithelial-myoepithelial carcinoma, Salivary gland, SEER, Surveillance epidemiology and end results, Population based

## Abstract

**Background:**

Epithelial-myoepithelial carcinoma is an uncommon malignant neoplasm seen most frequently in the salivary glands, representing approximately 1 to 2% of salivary gland tumors. Less than 600 cases have been reported in the literature since its initial description in 1972. The aim of this study was to examine demographic, site, stage, and survival factors in patients with epithelial-myoepithelial carcinoma.

**Methods:**

The 1973–2014 SEER (Surveillance, Epidemiology, and End Results) cancer database was queried for patients treated for epithelial-myoepithelial carcinoma. The data was analyzed for patient T (tumor), N (nodal), and M (metastasis) stage, tumor site, and demographic characteristics. The Kaplan-Meier model was used to estimate actuarial survival.

**Results:**

A total of 468 patients were identified. White patients represented 78.0% of the total. There were 291 female patients and 177 male patients. Overall 5-, 10-, and 20-year survival was 72.7%, 59.5%, and 38.3%, respectively. Mean survival time was 165.5 months. Parotid gland was the most common site with 57.7% of patients, with submandibular gland representing 9.8% of patients. Distant metastasis (M) status was unknown in 33.3%, with 2.6% being M1, 3.0% being MX, and 61.1% M0. Nodal metastasis (N) status was unknown in 33.3%, while 4.4% were N+, 4.7% were NX, and 57.5% were N0. 88.2% of patients had surgery as part or all of the treatment regimen. Univariate Kaplan-Meier analysis showed that AJCC overall stage, primary tumor (T) stage, nodal (N) stage, presence of distant metastasis (M1), age at diagnosis, race, and non-surgical treatment significantly affected survival. On multivariate analysis age, race, AJCC stage, T, N, M stage, and treatment type were significant.

**Conclusions:**

Epithelial-myoepithelial carcinoma is a malignant, histologically biphasic neoplasm most frequently seen in the parotid gland. The nodal and distant metastasis rates are low. Age at diagnosis, race, AJCC stage, T, N, M stage, and treatment type all significantly affected survival.

## Background

Epithelial-myoepithelial carcinoma is a relatively uncommon malignant neoplasm. It comprises approximately 1 to 2% of salivary gland neoplasms [1–3]. The histology of the tumor shows a biphasic pattern with clear myoepithelial cells encasing ducts with an epithelial lining. The tumor is typically regarded as a low-grade malignancy due to its low rate of nodal and distant metastasis [2]. In 2015 Vazquez et al. [2] examined the 1973–2010 SEER (Surveillance, Epidemiology, and End Results) database for patients with epithelial-myoepithelial carcinoma of the salivary glands. They noted 246 total cases with only 4.5% having distant metastases (M1). They noted an overall disease-specific survival (DSS) at 60 and 120 months of 91.3% and 90.2%, respectively. They noted that the majority of patients (95.7%) had surgery alone or in combination with another treatment such as radiation. The mean age was 63.8 years.

The goal of the present study was to update the SEER analysis for the 1973–2014 database, and to examine the factors influencing survival for patients with epithelial-myoepithelial carcinoma at all sites, including salivary primary sites. Demographic factors were examined and the Kaplan-Meier analysis was used to identify factors influencing survival in patients with epithelial-myoepithelial carcinoma.

## Methods

The SEER (Surveillance Epidemiology and End Results) database contains patient data from 1973 to 2014. The database was queried for malignant neoplasm, epithelial-myoepithelial carcinoma, any site, any age, and was extracted using SEER*Stat version 8.3.4 (National Cancer Institute, Bethesda, Maryland) and exported into Microsoft Excel 2016 (Microsoft Corporation, Redmond, Washington) for analysis. XLstat Biomed (Addinsoft, New York City, NY/Paris, France) was used for Kaplan-Meier overall survival analysis and log-rank analysis. Statistical significance was set at 0.05. SEER data was analyzed for overall survival, patient age, sex, and race, and tumor (T), nodal (N), and distant metastasis (M) stage, surgical treatment, and primary tumor site.

## Results

A total of 468 patients were identified. Table 1 illustrates the demographic data for age, sex, and race of the cohort. The majority of patients were > 50 years of age, with 65–69 years of age being the largest age group. There was a female predominance, with a 1.6:1 Female:Male ratio. The majority of patients with epithelial-myoepithelial carcinoma were white (78.0%), with black patients representing 11.1%, patients identified as other representing 9.8%, and race unknown in 1.1%. Table 2 illustrates the Tumor (T), Nodal (N), and Metastasis (M) characteristics of the cohort. The majority of the patients had T1 (21.8%) or T2 (21.6%) tumors. Of the patient cohort, 4.2% were N+, 57.5%were N0, 4.7% were NX (nodal status could not be determined) and 33.3% had an unknown nodal status. With respect to distant metastases, 61.1% were M0, 2.6% were M1, 5.2% were MX (metastatic status could not be determined), and 33.3% had an unknown metastatic status. Table 3 shows the primary site location of each patient. Parotid gland was the most common primary site, with 57.7%, followed by submandibular gland with 9.8%.

**Table 1:** Patient Demographics

Total	468	100%
Age, years		
10–14 years	2	0.4%
15–19 years	2	0.4%
20–24 years	2	0.4%
25–29 years	8	1.7%
30–34 years	7	1.5%
35–39 years	10	2.1%
40–44 years	15	3.2%
45–49 years	30	6.4%
50–54 years	50	10.7%
55–59 years	55	11.8%
60–64 years	44	9.4%
65–69 years	66	14.1%
70–74 years	60	12.8%
75–79 years	45	9.6%
80–84 years	35	7.5%
85+ years	37	7.9%
Sex		
Female	291	62.2%
Male	177	37.8%
Race		
White	365	78.0%
Black	52	11.1%
Other (American Indian/AK Native, Asian/Pacific Islander)	46	9.8%

**Table 2:** Patient tumor (T), nodal (N), and metastasis (M) characteristics

T		
T1	102	21.8%
T1a	3	0.6%
T1c	2	0.4%
T2	101	21.6%
T3	46	9.8%
T3a	1	0.2%
T3b	1	0.2%
T3c	6	1.3%
T3x	1	0.2%
T4	2	0.4%
T4a	13	2.8%
T4b	4	0.9%
TX	28	6.0%
TXa	2	0.4%
unknown	156	33.3%
N		
N0	269	57.5%
N1	16	3.4%
N1x	2	0.4%
N2	1	0.2%
N2NOS	1	0.2%
N3	1	0.2%
NX	22	4.7%
unknown	156	33.3%
M		
M0	286	61.1%
M1	12	2.6%
MX	14	5.2%
unknown	156	33.3%

**Table 3:** Patient primary site distribution

Primary site		
Abdominal esophagus	1	0.2%
Accessory sinus, NOS	1	0.2%
Anterior wall of nasopharynx	2	0.4%
Axillary tail of breast	1	0.2%
Base of tongue, NOS	2	0.4%
Breast, NOS	4	0.9%
Central portion of breast	2	0.4%
Cervical esophagus	1	0.2%
Cheek mucosa	5	1.1%
Conn, subcutaneous, other soft tis: lower limb, hip	1	0.2%
Conn, subcutaneous, other soft tis: trunk, NOS	2	0.4%
Conn, subcutaneous, other soft tis: upr limb, shoulder	1	0.2%
Endometrium	2	0.4%
Ethmoid sinus	1	0.2%
External ear	2	0.4%
Floor of mouth, NOS	2	0.4%
Hard palate	10	2.1%
Head, face or neck, NOS	1	0.2%
Lacrimal gland	1	0.2%
Lateral wall of nasopharynx	1	0.2%
Lower lobe, lung	3	0.6%
Lower-inner quadrant of breast	1	0.2%
Lower-outer quadrant of breast	1	0.2%
Lung, NOS	2	0.4%
Main bronchus	3	0.6%
Major salivary gland, NOS	14	3.0%
Maxillary sinus	5	1.1%
Middle lobe, lung	1	0.2%
Mouth, NOS	1	0.2%
Nasal cavity	6	1.3%
Nasopharynx, NOS	3	0.6%
Ovary	11	2.4%
Overlapping lesion of breast	5	1.1%
Overlapping lesion of floor of mouth	1	0.2%
Overlapping lesion of lung	1	0.2%
Overlapping lesion of major salivary glands	1	0.2%
Overlapping lesion of palate	1	0.2%
Overlapping lesion of rectum, anus, and anal canal	1	0.2%
Overlapping lesion of tongue	1	0.2%
Parotid gland	270	57.7%
Posterior wall of nasopharynx	2	0.4%
Pyriform sinus	1	0.2%
Retromolar area	1	0.2%
Sigmoid colon	1	0.2%
Skin of scalp and neck	2	0.4%
Skin other/unspec parts of face	1	0.2%
Soft palate, NOS	5	1.1%
Sublingual gland	1	0.2%
Submandibular gland	46	9.8%
Tonsillar fossa	1	0.2%
Trachea	3	0.6%
Unknown primary site	14	3.0%
Upper gum	1	0.2%
Upper lobe, lung	2	0.4%
Upper-inner quadrant of breast	2	0.4%
Upper-outer quadrant of breast	10	2.1%

**Fig. 1:**
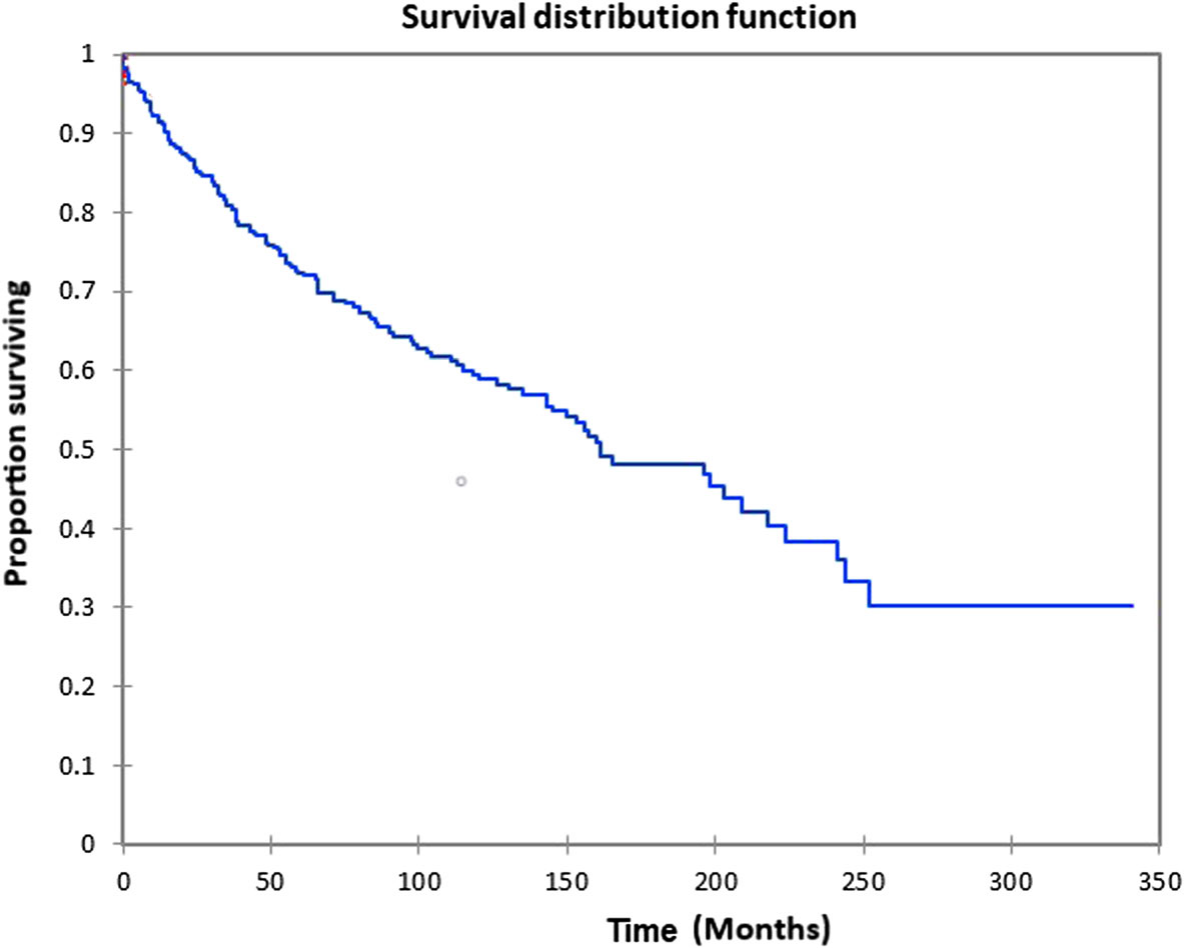
Kaplan-Meier overall survival for all patients

**Fig. 2:**
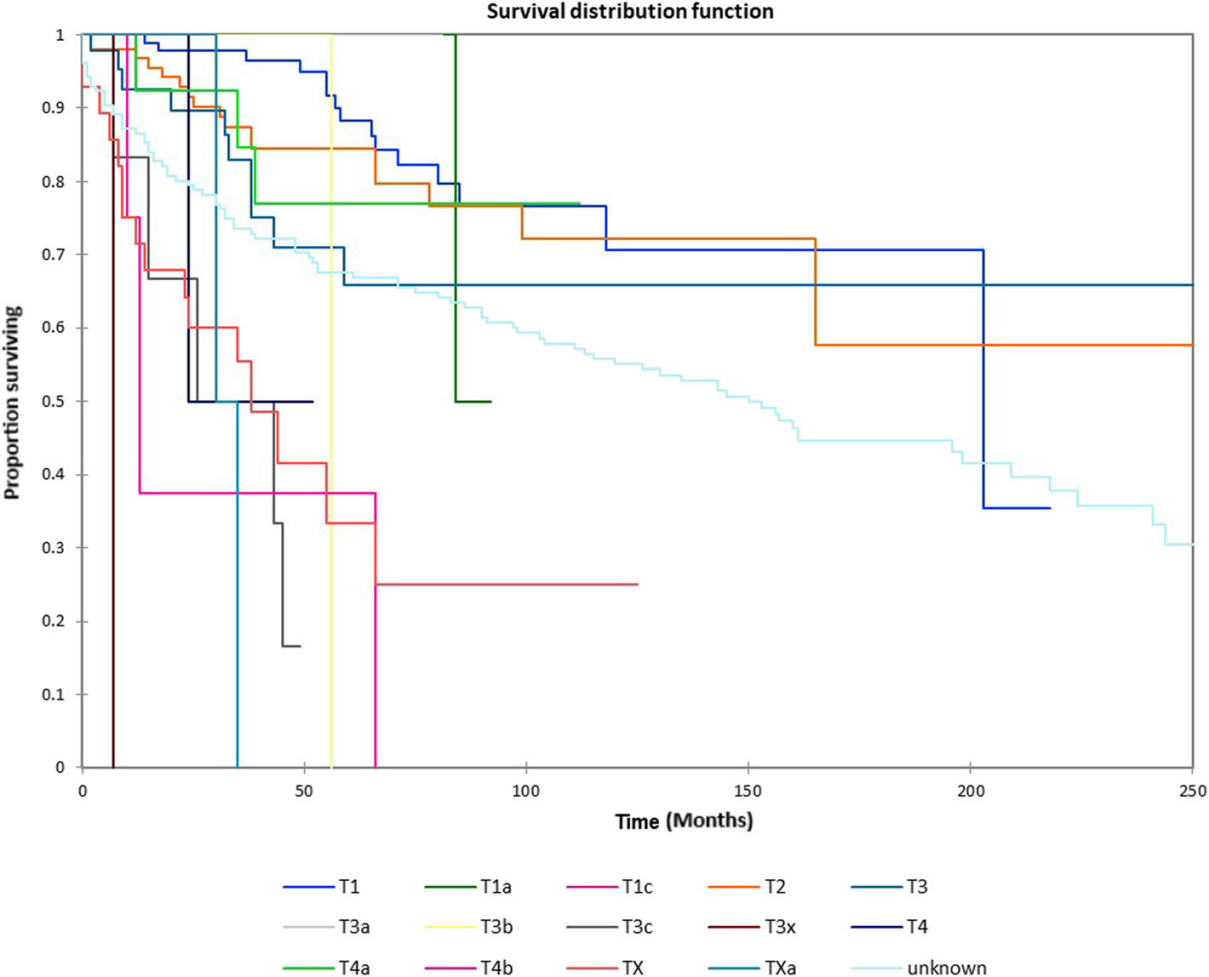
Kaplan-Meier overall survival by tumor (T) stage

**Fig. 3:**
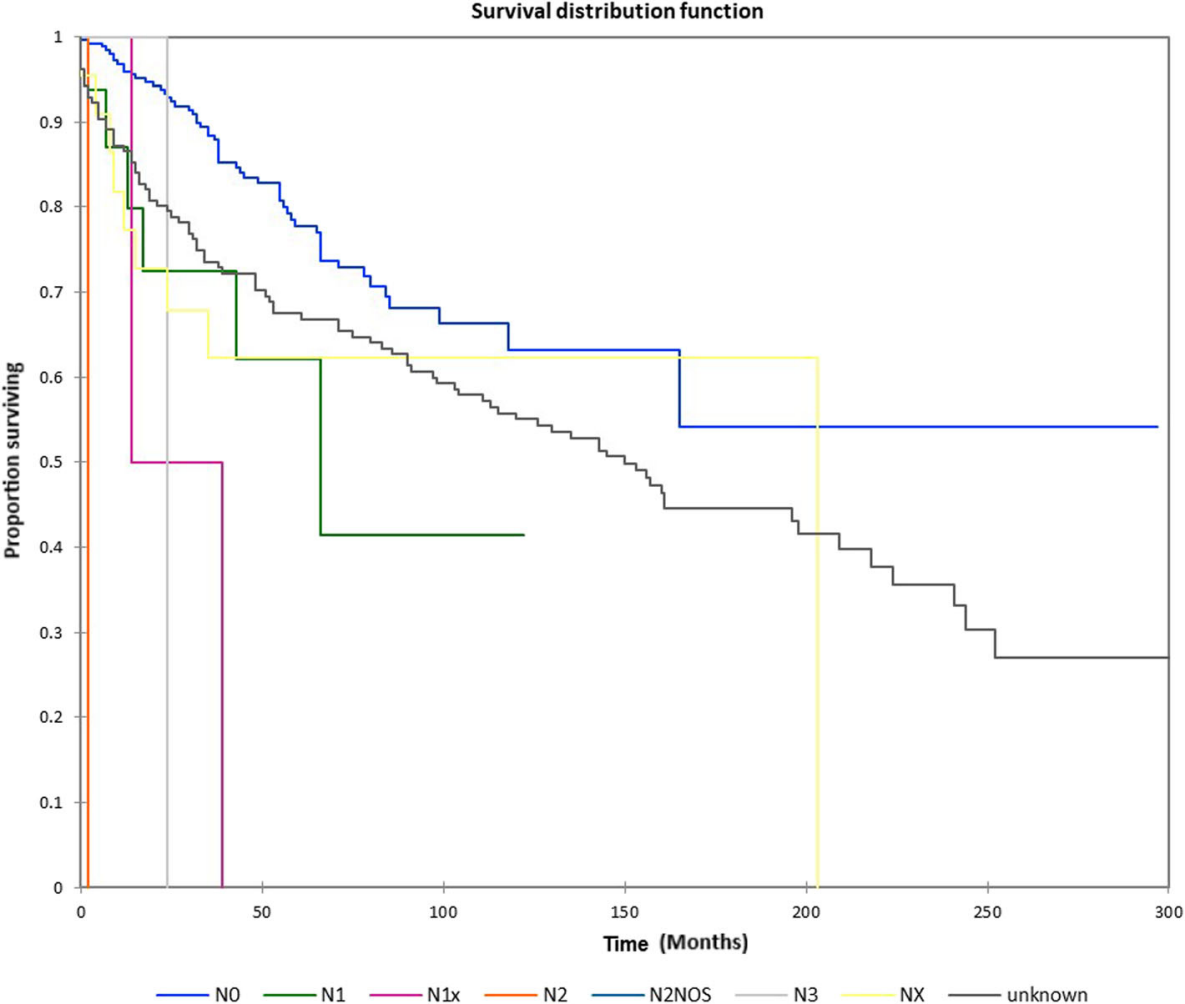
Kaplan-Meier overall survival by nodal (N) stage

**Fig. 4:**
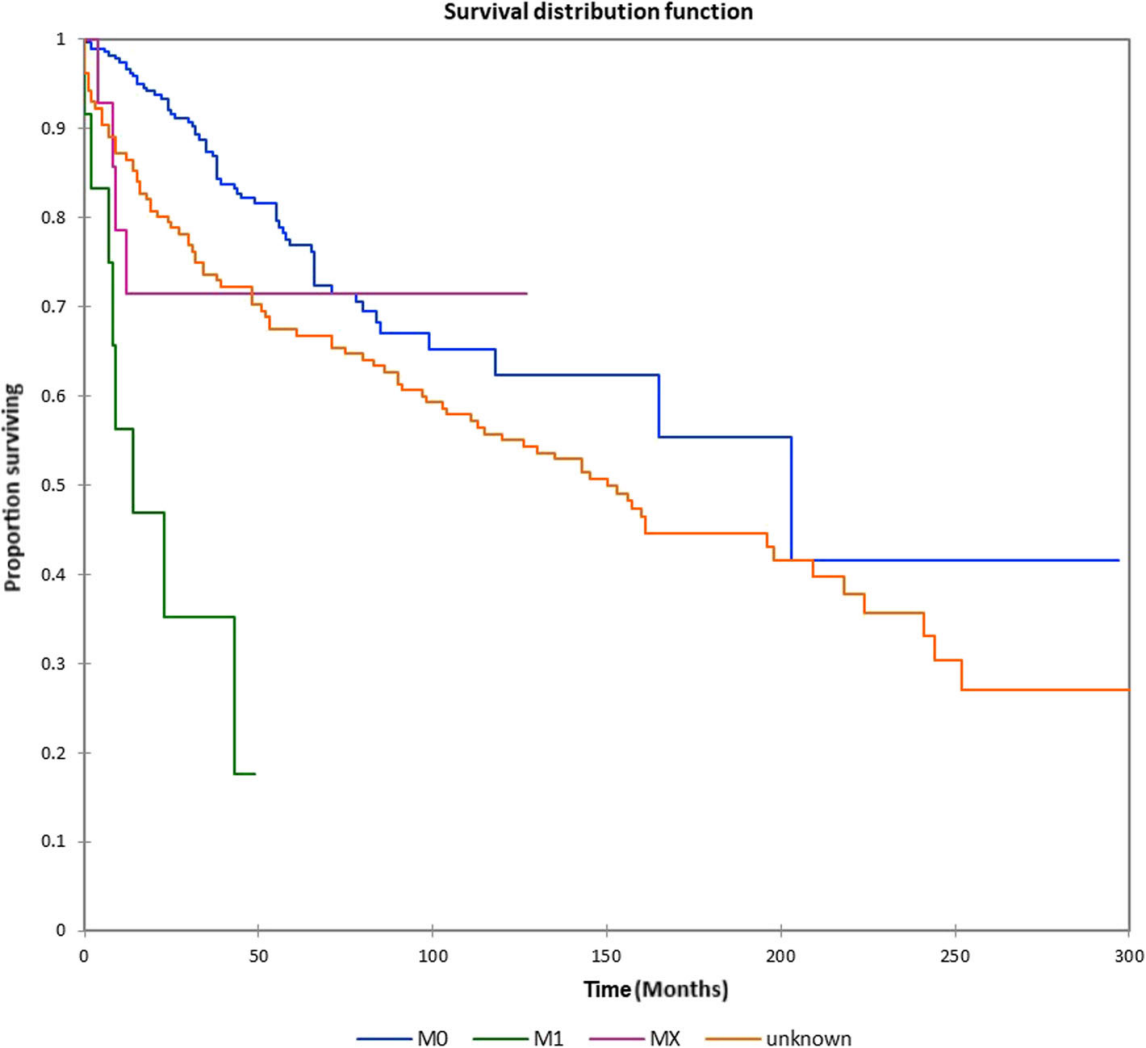
Kaplan-Meier overall survival by metastasis (M) stage

**Fig. 5:**
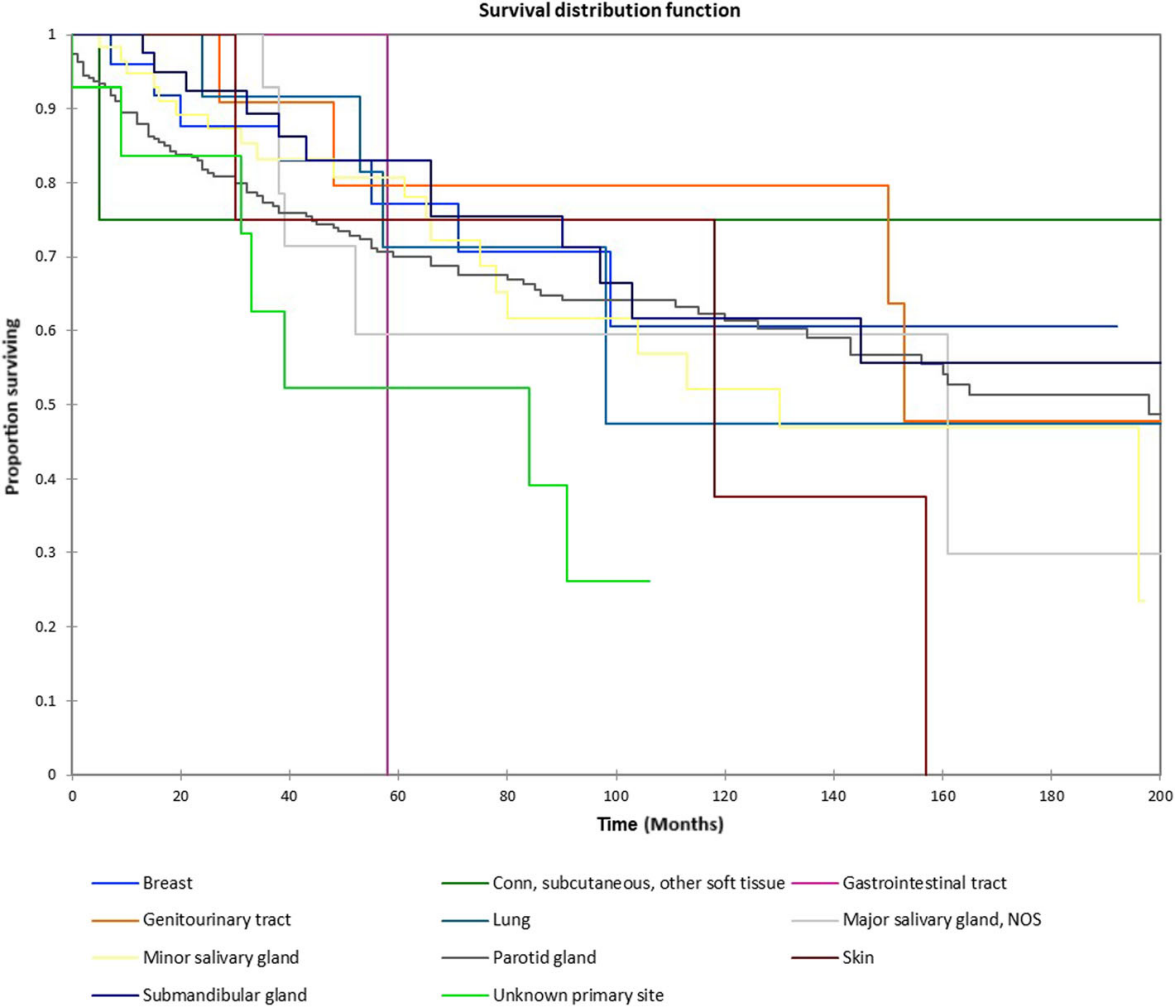
Kaplan-Meier overall survival by primary site

**Fig. 6:**
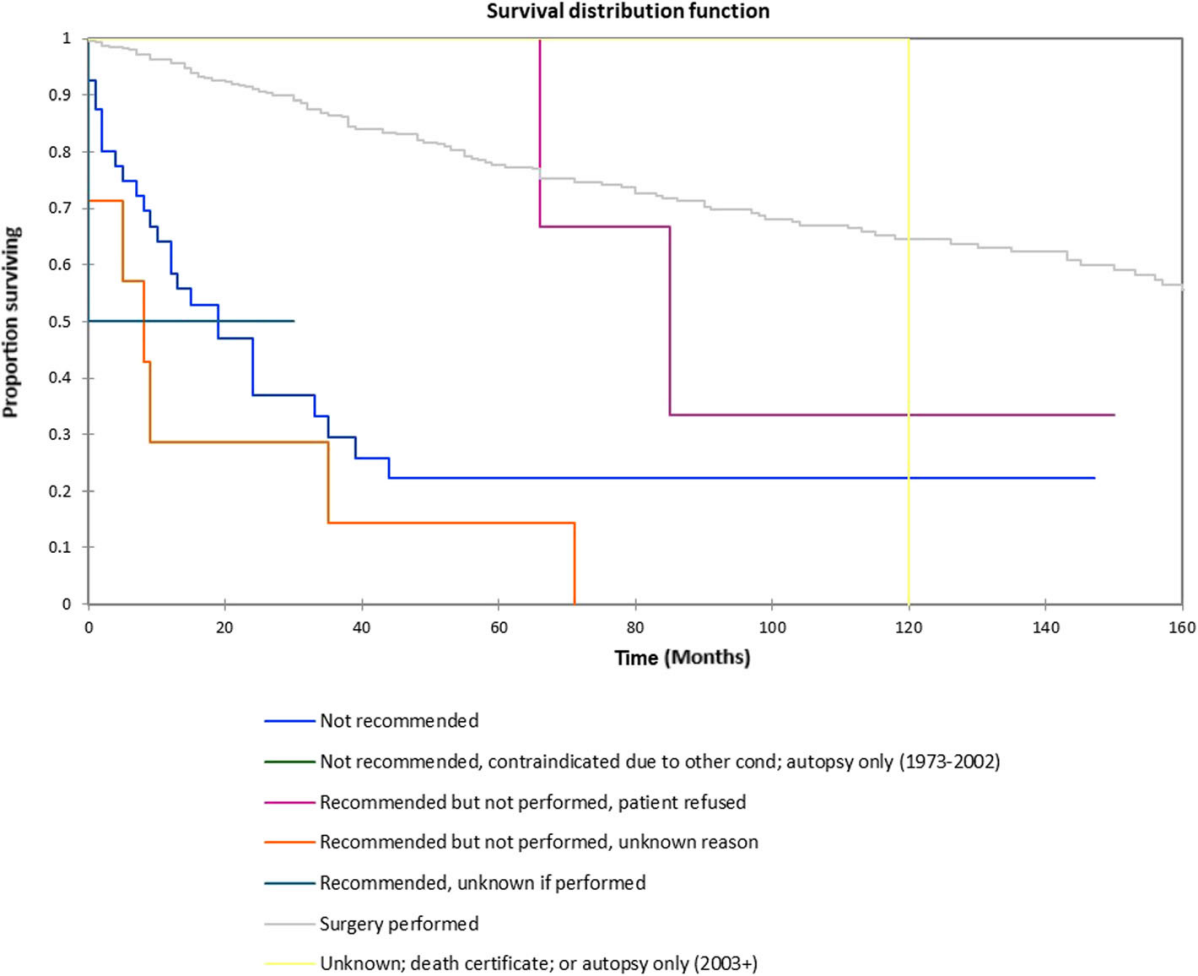
Kaplan-Meier overall survival by treatment

**Fig. 7:**
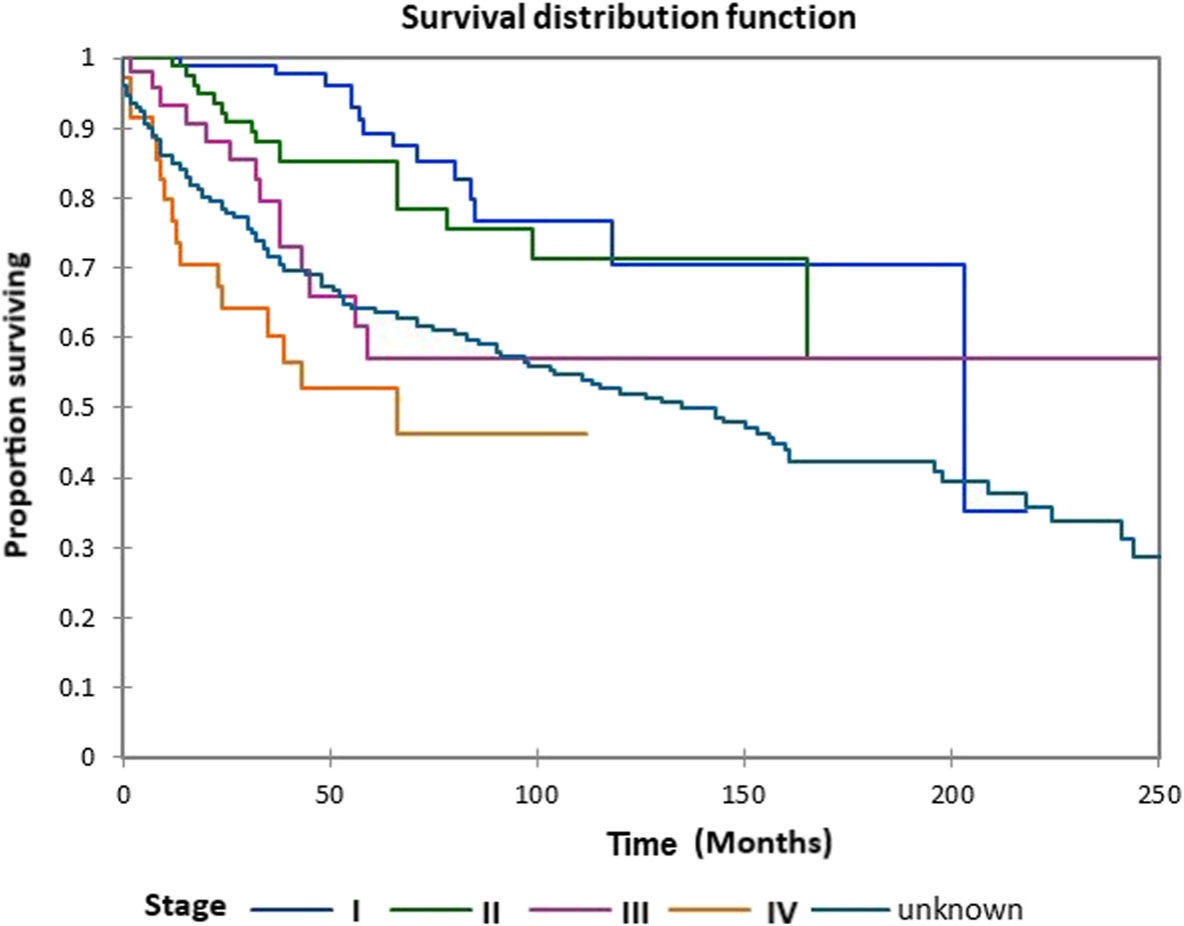
Kaplan-Meier overall survival by overall AJCC (American Joint Committee on Cancer) stage

**Fig. 8:**
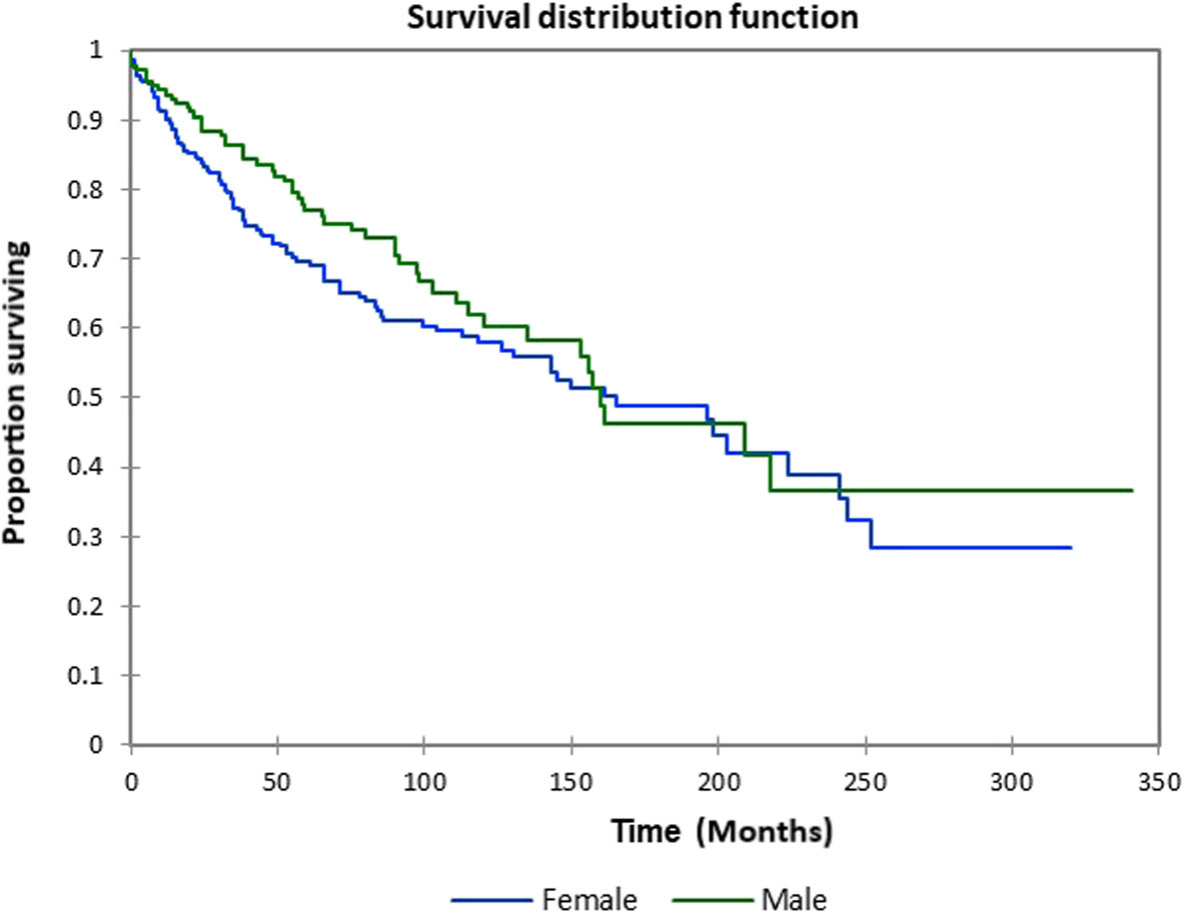
Kaplan-Meier overall survival by sex

**Fig. 9:**
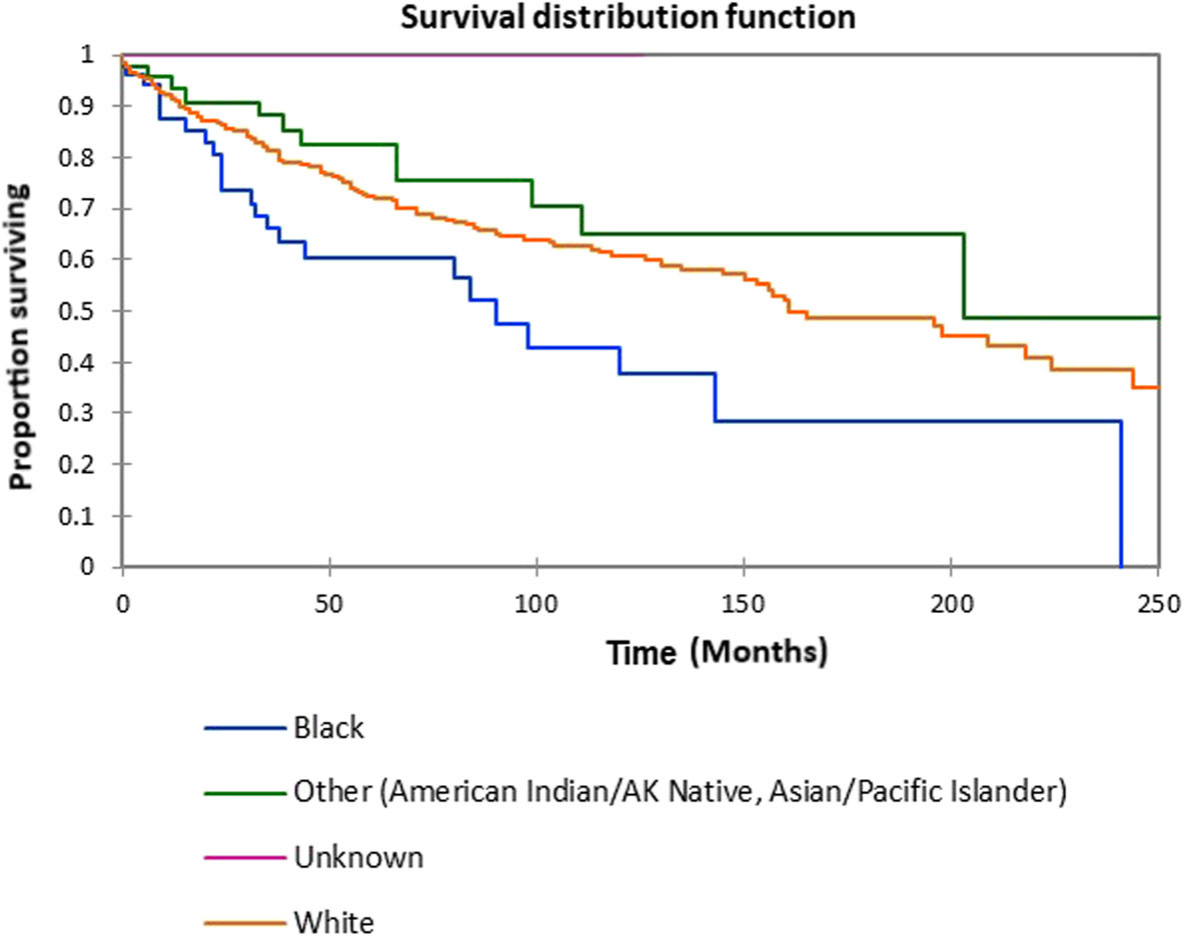
Kaplan-Meier overall survival by race

**Fig. 10:**
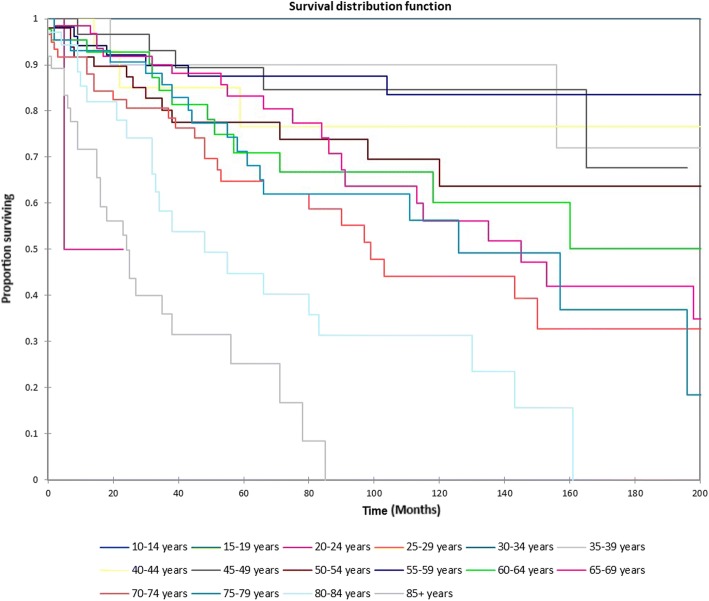
Kaplan-Meier overall survival by age at diagnosis

Figure 1 shows the Kaplan-Meier actuarial overall survival for the entire cohort. Overall survival at 5, 10, and 20 years was 72.7%, 59.5%, and 38.3%%, respectively. Figure 2 illustrates the Kaplan-Meier actuarial overall survival by T stage. Survival was lower for T2, T3, and T4 patients than for T1 patients (*p* < 0.0001). Figure 3 shows the Kaplan-Meier actuarial overall survival by N stage. Survival was significantly lower for N1, N2, and N3 patients than for N0 patients (*p* < 0.0001). Figure 4 shows the Kaplan-Meier actuarial overall survival by M stage. Survival for patients with no distant metastasis (M0) was significantly greater than M1 patients with distant metastasis present (*p* = < 0.0001). Figure 5 illustrates the Kaplan-Meier actuarial overall survival by primary site. There was no significant difference in survival for different primary sites (*p* = 0.6). Figure 6 illustrates the Kaplan-Meier actuarial overall survival by treatment regimen. Survival for patients on whom surgery was performed was significantly greater than patients for whom surgery was not recommended, patients for whom surgery was recommended but it was unknown whether the surgery was performed, and patients for whom surgery was recommended but not performed for an unknown reason (*p* < 0.0001). Figure 7 illustrates the Kaplan-Meier actuarial overall survival by overall AJCC (American Joint Committee on Cancer) stage. Survival decreased with increasing AJCC stage (I > II > III > IV, *p* < 0.0001). Figure 8 illustrates the Kaplan-Meier actuarial overall survival by sex. There was no significant difference in survival by sex (*p* = 0.2). Figure 9 illustrates the Kaplan-Meier overall survival by race. Survival was lower for black patients than for white or other (American Indian/AK (Alaska) Native, Asian/Pacific Islander), *p* = 0.01). Figure 10 illustrates the Kaplan-Meier overall survival by age at diagnosis. Survival was significantly lower for patients over age 80 at diagnosis (*p* < 0.0001). Multivariate logistic regression analysis was performed. This demonstrated that age (*p* < 0.0001), race (*p* = 0.02), AJCC stage (*p* < 0.0001), T (*p* = 0.006), N (*p* < 0.0001), and M (*p* = 0.003) stage, and treatment type (*p* < 0.0001) were significantly associated with survival. Primary tumor site and patient sex were not significantly associated with survival on multivariate analysis.

## Discussion

Epithelial-myoepithelial carcinoma is an uncommon biphasic neoplasm most commonly arising in salivary tissue. It represents approximately 1% of all salivary neoplasms [2, 3]. In their review of 246 cases of salivary gland epithelial-myoepithelial carcinoma from the 1973–2010 SEER database, Vazquez et al. [2] noted that 57.3% of patients were female. The present study noted a similar female predominance, with 62.2% of patients being female. Vazquez et al. noted a rate of distant metastases at diagnosis of 4.5%. The present cohort shows an even lower M1 rate of 2.6%. Overall disease-specific survival at 60 and 120 months in the Vazquez study was 91.3% and 90.2%. The present study showed a lower overall survival of 72.7% and 59.5% at 60 and 120 months. This may be due to the inclusion in the present study of all sites including unknown primary, lung, and gastrointestinal tumors. These sites were less prevalent than salivary primary sites such as parotid and submandibular gland but had lower mean survival times, likely leading to the lower overall survival for the entire cohort. Additionally, the updated 1973–2014 data included in the present study showed a lower overall survival for parotid epithelial-myoepithelial carcinomas than submandibular and minor salivary gland tumors. In the 2015 study [2] patients with low-grade histology and lesions < 4 cm had significantly better survival vs. patients with high-grade histology and tumors > 4 cm. In the present study univariate analysis showed that T stage, N stage/presence of nodal metastases, M stage/presence of distant metastases, surgical treatment vs. nonsurgical treatment, overall AJCC stage, race, and age at diagnosis all significantly affected survival. Specifically, patients with T2, T3, and T4 tumors showed significantly shorter survival than patients with T1 tumors, patients with M1 tumors showed significantly shorter survival than M0 patients, and patients with nodal metastases showed significantly shorter survival than patients who were N0. Chen et al. [1] reported a rare case of a patient with a primary base of tongue epithelial-myoepithelial carcinoma with neck metastases and multiple lung nodules (T4N2cM1). The patient refused treatment other than biopsy and expired at 18 months post-diagnosis. In the present cohort parotid and submandibular gland were the two most common primary sites, but a statistically significant difference in survival by primary site was not found in the present study. Patients treated with surgery showed significantly increased survival relative to patients in whom surgery was not recommended, recommended but not performed for an unknown reason or in patients for whom surgery was recommended but it was unknown whether surgery was actually performed. Additionally, black patients and patients age 80 or greater at time of diagnosis had significantly shorter survival than other patients. These findings remained significant on multivariate logistic regression analysis, with overall AJCC stage, T, N, and M stage, age > 80, black race, and nonsurgical treatment significantly affecting survival.

It is interesting to note that between 1973 and 2010 there were 246 salivary gland epithelial-myoepithelial carcinomas reported (an average of approximately 6.6 per year), while in the updated data in the present study a further 70 epithelial-myoepithelial carcinomas of the major salivary glands (parotid + submandibular gland) were recorded in the next 4 years from 2010 to 2014 (an average of approximately 17.5 per year). This relative increase in numbers from 2010 to 2014 may be due to several factors. One significant factor may be Hurricane Katrina. Hurricane Katrina struck the Gulf Coast of the United States from August 23 to August 31, 2005 and significantly affected central Florida to Texas. These areas contained several major hospitals, and the SEER database specifically notes in their data set that Hurricane Katrina significantly impacted the Louisiana Cancer Registry’s data reporting for the second half of 2005, and SEER excluded Louisiana cases diagnosed from July–December 2005. As this period is contained within the 1973–2010 period, this may have had some effect on the epithelial-myoepithelial carcinoma cases reported during this time period. Additionally, increased familiarity by pathologists with epithelial-myoepithelial carcinoma may have led to increased histopathological identification of this rare tumor, and decreased misdiagnosis as more common neoplasms such as mucoepidermoid carcinoma or adenoid cystic carcinoma. Finally, the increased availability and use of imaging modalities such as ultrasound, computed tomography, and magnetic resonance imaging even at smaller regional centers may have led to both increased diagnostic sensitivity for epithelial-myoepithelial carcinomas presenting as neck masses, etc., and may have also led to increased discovery of epithelial-myoepithelial carcinomas as “incidentalomas” discovered on diagnostic imaging ordered for other, unrelated reasons.

This study has several limitations. The retrospective data makes recall bias and selection bias a possibility, and the reporting limitations of the SEER database meant that a minority of the cohort had missing TNM/overall stage data. However, SEER data has been frequently used to evaluate factors affecting patient survival in rare tumors for which randomized prospective trials would be difficult to assemble [2, 4, 5]. The survival analysis by treatment may be confounded by co-morbidities in some patients, particularly patients in whom surgery was not recommended.

## Conclusions

Epithelial-myoepithelial carcinoma is an uncommon biphasic malignancy most commonly seen in the parotid and submandibular glands. The rate of nodal and distant metastases is low; less than 5% for either in the present study. The tumor typically shows a low-to-intermediate grade behavior, with 5-, 10-, and 20-year overall survival of 72.7%, 59.5%, and 38.3% in the present study, and a mean survival time of 165.5 months. Surgical treatment appears to be the mainstay of treatment, with radiation reserved for positive or close margins or patients who are not surgical candidates or who refuse surgery. Lower T stage, absence of regional nodal or distant metastases, age < 80 years at diagnosis, white or Asian/pacific islander/native American race, lower overall AJCC stage, and surgical treatment all appeared to significantly affect overall survival.

## Abbreviations

AJCC: American Joint Committee on Cancer
BOT: Base of tongue
M: Metastasis
N: Nodal
NOS: Not otherwise specified
OS: Overall survival
SEER: Survival, Epidemiology, and End Results
T: Tumor
